# Association of Leucine-Rich Glioma Inactivated Protein 1, Contactin-Associated Protein 2, and Contactin 2 Antibodies With Clinical Features and Patient-Reported Pain in Acquired Neuromyotonia

**DOI:** 10.1001/jamaneurol.2018.2681

**Published:** 2018-09-17

**Authors:** Angela Vincent, Philippa Pettingill, Rosie Pettingill, Bethan Lang, Ron Birch, Patrick Waters, Sarosh R. Irani, Camilla Buckley, Osamu Watanabe, Kimiyoshi Arimura, Matthew C. Kiernan

**Affiliations:** 1 Nuffield Department of Clinical Neurosciences, University of Oxford, John Radcliffe Hospital, Oxford, United Kingdom; 2 Patient representative, Highland Park, Gold Coast, Australia; 3 Department of Neurology and Geriatrics, Kagoshima University Graduate School of Medical and Dental Sciences, Kagoshima, Japan; 4 Brain and Mind Centre, University of Sydney, Sydney, Australia; 5 Department of Neurology, Royal Prince Alfred Hospital, Sydney, Australia

## Abstract

**Importance:**

Although acquired autoimmune neuromyotonia (NMT) is associated with voltage-gated potassium channel (VGKC)–complex antibodies, to date there has been no systematic study of autoantibodies to the specific antigens leucine-rich glioma inactivated protein 1 (LGI1), contactin-associated protein 2 (CASPR2), and contactin 2 together with the full clinical syndrome, particularly pain and autonomic and central nervous system involvement.

**Objectives:**

To study the full spectrum of clinical features and serum autoantibodies in patients with NMT, including the effects of pain on quality of life.

**Design, Setting, and Participants:**

A cohort study of clinical features and serologic testing in 38 patients with electrophysiologically-confirmed NMT, reviewed clinically between February 2007 and August 2009, in the Universities of Sydney and Kagoshima and followed up across 2 to 4 years. Association of NMT with quality of life was researched in an independent, patient-led, online pain survey conducted from April 2012 to May 2012. Serologic analyses were performed in 2012, and final data analysis was performed in 2016.

**Main Outcomes and Measures:**

Clinical data and scores on the modified Rankin Scale (mRS), which measures disability on a range of 0 to 6, with 0 indicating normal and 6 indicating death, before and after treatments were combined with CASPR2, LGI1, and contactin 2 antibody status.

**Results:**

Among the 38-person NMT cohort, 25 (65.8%) were male and the median (range) age was 55 (12-85) years. Twenty-three (60.5%) were Japanese and 15 (39.5%) were of white race/ethnicity. Symptomatic treatments (mainly antiepileptic drugs) were used in most patients with mild disease (12 patients with mRS <3), whereas immunotherapies were successful in most patients with mRS scores greater than 2. Autoantibodies to VGKC-complex antigens (17 patients [45%]), bound to CASPR2 (5 [13%]), contactin 2 (5 patients, 1 with CASPR2 [13%]), LGI1 (2 [5%]), or both LGI1 and CASPR2 (6 [16%]). The last group of 6 patients had high mRS scores (mean [SD], 3.8 [1.7]), thymoma (4 patients), pain (5 patients), autonomic (6 patients) and sleep (5 patients) disturbance, suggesting Morvan syndrome. The 56 responders to the independent patient-led survey reported pain that could be severe, anatomically widespread, and that often resulted in unemployment, domestic problems, and poor quality of life.

**Conclusions and Relevance:**

The cohort study detailed underrecognized aspects of the clinical and serologic spectrum of NMT. The heterogeneity of clinical features and of specific antibodies limit associations, but the common existence of thymoma, pain, and autonomic and central nervous system features, often with both LGI1 and CASPR2 antibodies, should be better recognized to more completely address the range of comorbidities and consequences of the disease regarding quality of life.

## Key Points

**Questions:**

Which specific targets of voltage-gated potassium channel–complex antibodies are found in acquired neuromyotonia, and are these antibodies associated with additional clinical features of tumors, pain, or autonomic or central disorders?

**Findings:**

This cohort study combined with a patient-led survey found that antibodies to the extracellular aspects of leucine-rich glioma inactivated protein, contactin-associated protein 2, and contactin 2 were variably present in 45% of patients with neuromyotonia. Paresthesia and various pain manifestations were common in neuromyotonia, and the type and severity of pain were found to exert a substantial influence on quality of life.

**Meaning:**

Antibodies to voltage-gated potassium channel–complex proteins are not found in all patients with neuromyotonia and do not individually relate to specific clinical features, but the presence of pain and its effects on quality of life need greater recognition.

## Teaser

This combined clinical cohort and survey study examines associations between neuromyotonia and voltage-gated potassium channel–complex–specific antibodies and the consequences of neuromyotonia for quality of life.

## Introduction

Peripheral nerve hyperexcitability syndromes include neuromyotonia or Isaacs syndrome and can be genetic or acquired. Acquired neuromyotonia (NMT) was first associated with antibodies that immunoprecipitated voltage-gated potassium channels (VGKCs).^1,2,3^ Voltage-gated potassium channel antibodies were then identified in patients with the rare Morvan syndrome^4^ and in a form of limbic encephalitis.^5,6,7^ Subsequently, it was shown that these antibodies were not directed against the extracellular domains of VGKCs but to 3 proteins tightly complexed with the VGKCs in detergent extracts of mammalian brain tissue: leucine-rich glioma inactivated protein 1 (LGI1), contactin-associated protein 2 (CASPR2), and contactin 2.^8^ Antibodies to LGI1 were strongly associated with limbic encephalitis, whereas antibodies to CASPR2 were found more often in patients with Morvan syndrome or NMT,^8,9,10,11^ sometimes with LGI1.^8,11^ Contactin-associated protein 2 antibodies were associated with underlying thymomas,^12^ but contactin 2 antibodies were uncommon.^8^

The reported frequency of VGKC-complex antibodies in patients with NMT, usually at relatively low titers (100-400pM), or in those with the less severe variant, cramp fasciculation syndrome, has varied between 2% and 40%.^13,14,15,16^ Other studies have included only a few patients with NMT.^8,10,17^ The full spectrum of clinical features (including pain, which has not been well studied previously), the specific antigenic targets, and whether the clinical features relate to antibody specificity have not been determined in NMT.

We describe detailed clinical and serologic characteristics of patients with NMT from Japan and Australia combined with the results of a novel, independent, patient-led pain questionnaire sent to individuals registered on an online forum for persons with Isaacs syndrome. The study is particularly timely given recent evidence that injection of patient-derived CASPR2 antibodies or genetic deletion of CASPR2 causes afferent nerve hyperexcitability and mechanical allodynia in mice.^18^

## Methods

Thirty-eight serum samples from patients with clinical and neurophysiologic features consistent with a diagnosis of NMT were collected in Sydney, Australia,^19^ after VGKC-complex antibody screening, and Kagoshima, Japan, between February 2007 and August 2009 for routine testing at the time of patient review and were studied in detail in 2012. Final data analysis was performed in 2016. The diagnosis was reached according to established criteria, with symptoms or signs of muscle twitching or muscle cramps affecting at least 2 regions of skeletal muscles.^14^ All patients demonstrated the characteristic electromyographic (EMG) discharges consisting of doublet, triplet, or multiplet single–motor unit discharges with a high intraburst frequency of between 40 and 400 per second.^3,20,21^ Eleven patients with similar symptoms and serologic results, but without confirmation or testing by EMG, were excluded. Ethical approval was granted by the South Eastern Sydney Local Health District (Sydney, Australia), the University of Sydney (Sydney), and the University of Kagoshima (Kagoshima, Japan) human research ethics committees, and patients gave written informed consent for their data to be used. The serologic study was performed under the authority of the Oxford Regional Ethics Committee.

### Antibody Tests

Serum samples were originally tested for binding to VGKC-complex antibodies by radioimmunoprecipitation, as previously described.^3,8^ Antibodies to LGI1, CASPR2, and contactin 2 were detected by live cell–based assays. For these assays, DNA encoding the different proteins was transfected into human embryonic kidney 293 cells cultured on glass coverslips and left overnight at 37°C. The cells were gently washed and left in medium for 1 additional day before each coverslip was placed in a 20-well microtiter plate and incubated in 200 μL of medium containing 10 μL (1:20 for LGI1 or contactin 2) or 2 μL (1:100 for CASPR2) for 2 hours. Binding of IgG to the cells was detected with Alexa fluor anti-human IgG (Thermo Fisher Scientific) after fixation.^8^ The binding was scored visually on a scale from 0 (negative), 1 (low positive), and 2 to 4 (increasing strength of binding).

### Clinical Information and Pain Questionnaire

The clinical data recorded (shown in Table 1; data requested are shown in eTable 1 in the Supplement) covered symptom history and symptoms at first clinic visit and at neurologic review, with specific reference to pain, autonomic symptoms, central nervous system features, tumors, investigations (all patients underwent EMG; magnetic resonance imaging, electroencephalogram, or cerebrospinal fluid abnormalities were documented if available), and modified Rankin Scale (mRS) scores at first review and follow-up, which was 2 to 4 years after review. The mRS measures disability on a range of 0 to 6, with 0 indicating normal and 6 indicating death.

**Table 1.** Presenting Features and Neurophysiologic Findings in 38 Patients With Neuromyotonia

Feature	No. (%)		
	Total (N = 38)	Male (n = 25)	Female (n = 13)
Age, median (range), y	55 (12-85)	55 (12-85)	44 (25-79)
EMG features defining NMT			
Multiplets	19 (50)	14 (56)	5 (38)
Fasciculations	10 (26)	6 (24)	4 (31)
Doublets	8 (21)	6 (24)	2 (15)
Myokymic discharges	7 (18)	4 (16)	3 (23)
Neuromyotonic discharges	7 (18)	3 (12)	4 (31)
Bursts	6 (16)	5 (20)	1 (8)
Repetitive discharges	3 (8)	0	3 (23)
Symptoms reported on referral			
Cramps	16 (42)	13 (52)	3 (23)
Fasciculations	7 (18)	5 (20)	2 (15)
Myokymia	4 (11)	1 (4)	3 (23)
Spasms	3 (8)	3 (12)	0
Stiffness	4 (11)	2 (8)	2 (15)
Fatigue	1 (3)	0	1 (8)
Paresthesia or pain	3 (8)	2 (8)	1 (8)
Altered sensation	1 (3)	0	1 (8)
Features in response to specific questions			
Cramps	32 (84)	22 (88)	10 (77)
Twitching	30 (79)	20 (80)	10 (77)
Sweating	12 (32)	9 (36)	3 (23)
Weakness	13 (34)	10 (40)	3 (23)
Stiffness	12 (32)	9 (36)	3 (23)
Pseudomyotonia	7 (18)	4 (16)	3 (23)
Autonomic disturbance			
Any	18 (47)	12 (48)	6 (46)
Constipation	4 (11)	3 (12)	1 (8)
Diarrhea	3 (8)	3 (12)	0
Excessive secretions, including sweating	8 (21)	4 (16)	4 (31)
Tachycardia or tachypnea	4 (11)	2 (8)	3 (23)
Other (1 each of dry mouth, hypothermia, hyperthermia, gastrointestinal, and erectile dysfunction)	5 (13)	4 (16)	1 (8)
Sensory features			
Pain or paresthesia or both	20 (53)	14 (56)	6 (46)
CNS features			
Any	11 (29)	9 (36)	2 (15)
Neuropsychiatric features of agitation or anxiety	10 (26)	8 (32)	2 (15)
Insomnia	10 (26)	8 (32)	2 (15)
Other sleep disturbance	3 (8)	2 (8)	1 (8)
Depression	5 (13)	4 (16)	1 (8)
Cognitive problems	1 (3)	0	1 (8)
Seizures	2 (5)	1 (Previous)	1 (8)
Tumor types	8 Concurrent, 2 previous (26)	3 Thymomas, 3 prostates, 1 previous non–small cell lung cancer, 1 previous leukemia (32)	2 Thymomas (15)

Abbreviations: CNS, central nervous system; EMG, electromyography; NMT, neuromyotonia.

A patient-led, online pain questionnaire was initiated through a patient support network from April 2012 to May 2012, and individual patients were contacted by a patient representative (R.B.) through http://isaacsyndrome.proboards.com/ (now https://www.facebook.com/groups/isaacs.pnh/) and asked whether they had pain. One hundred seventy-six patients responded, of whom 165 reported pain and were sent the questionnaire (eTable 2 in the Supplement); full responses were obtained from 56 patients, deidentified by one of us (R.B.), and analyzed by another of us, a neuroimmunologist (A.V.).

### Statistical Analysis

Prism software, version 7 (GraphPad Software) was used to create graphs and perform statistical analysis. Two-tailed paired *t* tests were used to compare scores before and after treatments, and a 2-sided *P* < .05 was considered significant.

## Results

Of the 38 patients, 25 (66%) were male and the median (range) age was 55 (12-85) years. Twenty-three (60.5%) were Japanese and 15 (39.5%) were of white race/ethnicity. The cohort was defined by typical history and EMG findings of peripheral nerve hyperexcitability. Nerve conduction was normal in 10 of 12 patients examined; 2 patients had evidence of neuropathy (1 polyneuropathy; 1 sensory more than motor).

### Clinical Symptoms of Patients With Diagnosed NMT

Initial presenting symptoms on referral comprised cramps (16 patients [42%]), fasciculations (7 [18%]), and, less commonly, symptoms of myokymia, stiffness, or spasms (Table 1). Sensory features were reported by 8 patients (21%). Possible precipitating events were recorded by 2 patients (1 “seafood poisoning and vomiting,” 1 “infection and exhaustion”).

The symptoms recorded in response to specific questions at the first clinic visit were more widespread, including cramps (32 [84%]) and muscle twitching (30 [79%]), as well as sweating (12 [32%]), weakness (13 [34%]), and stiffness (12 [32%]) (Table 1). In addition, autonomic disturbance involving excessive secretions, sweating, diarrhea, tachycardia, and tachypnea was evident in 18 patients (47%) (Table 1). One patient had evidence of orthostatic hypotension.

The most striking additional complaint reported was sensory disturbance. Specifically, 20 patients (53%) complained of paresthesia (5 [13%]), pain (8 [21%]), or both (7 [18%]). Pain and paresthesia were typically reported in the legs or arms and sometimes in all limbs. Pain was described as burning or throbbing in 7 patients (18%). One patient specifically reported pain from muscle cramps.

With respect to central nervous system symptoms, sleep disturbance, particularly insomnia, was present in 13 patients (34%), anxiety and agitation in 10 (26%), and depression in 5 (13%). Two male patients (5%) attempted suicide. One patient had new-onset seizures.

Additional investigations included cerebrospinal fluid analysis in 8 patients, which had normal results with the exception of 2 patients (25%) who had a small increase in lymphocyte counts. Electroencephalogram results were normal in 8 of 9 patients (89%) (1 woman with a locally invasive thymoma had focal spikes and epilepsy with normal findings on magnetic resonance imaging), and normal findings on magnetic resonance imaging in 18 of 20 others (90%) (1 had degenerative spinal changes and 1 had temporal lobe atrophy of unknown cause).

### Other Autoimmune Disorders or Tumors

Eight of the 38 patients (21%) had recent tumors, 5 thymomas (3 males, 2 females), and 3 prostate tumors; 1 had a previously treated non–small cell cancer; and 1 had acute lymphatic leukemia after bone marrow transplantation. Acetylcholine receptor antibodies were positive in 3 patients with thymoma and Hu antibodies in another patient with thymoma.

Overall, autoimmunity or other comorbidities (eTable 3 in the Supplement), mainly diabetes or hypertension, were reported in 20 patients (53%). Antinuclear antibodies were present in 4 patients and anti–thyroid peroxidase antibodies in 1.

### Treatments

**Figure 1.**
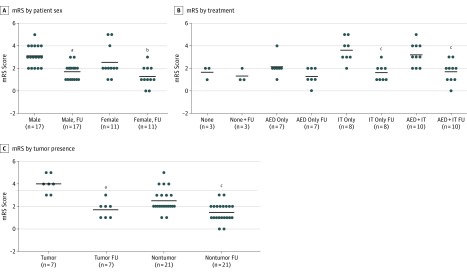
Modified Rankin Scale (mRS) Scores in 28 Patients With Neuromyotonia (NMT) Before and After Treatment^a^ A, Scores in male and female patients before treatment and at follow-up (FU). B, Scores before treatment and at FU according to treatment type. Further information on treatment responses is given in eTable 4 in the Supplement. C, Scores in patients with tumors (3 thymomas, 3 prostate tumors, and 1 acute lymphocytic leukemia after bone marrow transplant) and without tumors. In most cases, mRS scores were lower at FU (2-tailed paired *t* tests). The mRS measures disability on a range of 0 to 6, with 0 indicating normal and 6 indicating death. Solid horizontal lines indicate means. *P* values were determined by 2-tailed *t* test. AED indicates antiepileptic drugs and other symptomatic therapies; IT, immunotherapy. ^a^*P* < .001 for the change in mRS score at FU. ^b^*P* = .02 for the change in mRS score at FU. ^c^*P* = .001 for the change in mRS score at FU.

Modified Rankin Scale scores and information on treatments used were available for 28 patients. The scores were variable but similar between male and female patients and generally improved after treatment (Figure 1A), but patients with mild disease who were untreated (n = 3) or given treatment for symptoms only (2 received phenytoin; 4, carbamazepine; and 1, clonazepam plus diazepam) had insignificant benefits (Figure 1B). The remaining 18 patients had more severe disease (mean [SD] mRS score, 3.39 [1.04] vs 2.0 [0.81]; *P* = .001) and had received a range of immunotherapies only or in combination with drugs for symptoms, with clear benefits in most (Figure 1B). The 7 patients with tumors had more severe disease (mRS, 3.75 [1.04]) than the 21 patients without tumors mRS 2.5 [0.98], *P* = .004) but also responded well to treatments (Figure 1C). Despite the improvements, in a number of patients specific drugs were judged unhelpful (a summary of treatment responses is provided in eTable 4 in the Supplement).

### Autoantibodies and Clinical Associations

**Figure 2.**
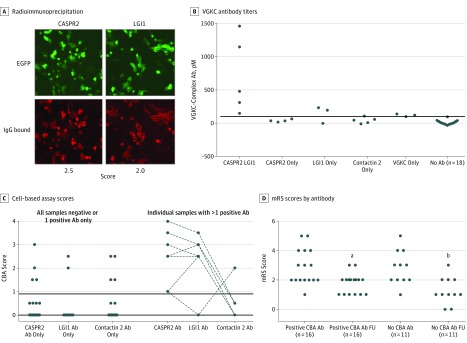
Antibodies in Patients With Neuromyotonia (NMT) A, A serum sample positive for both contactin-associated protein 2 (CASPR2) and leucine-rich glioma inactivated protein 1 (LGI1) antibodies. The binding of the patient’s IgG antibodies (Abs) to human embryonic kidney cells (enhanced green fluorescent protein [EGFP] [green] label) was detected with Alexa fluor anti-human IgG (red). Binding was scored on a scale of 0 to 4, with 0 indicating negative; 1, positive; and 2 to 4, increasing positivity. This serum sample scored 2.5 and 2.0 as shown. Results of tests for contactin 2 Abs were negative (data not shown). This patient had mild disease (modified Rankin Scale [mRS] score, 2), unlike the other 5 patients with LGI1 and CASPR2 Abs (mRS scores, 3-5), and his disease responded to carbamazepine with immunotherapies (mRS score, 1). B, Voltage-gated potassium channel (VGKC)–complex antibody titers associated with the presence of specific Abs or none. The horizontal line indicates the cutoff for positivity, 1. Eleven serum samples were positive (>100pM) by radioimmunoprecipitation, but high titers were only found in those with LGI1 and CASPR2 Abs. C, The cell-based assay (CBA) scores in patients with either a single or 2 different Ab specificities. The horizontal line indicates the cutoff for positivity, 100pM. CASPR2 and LGI1 antibodies together gave the highest binding scores, but few reached the maximum score of 4. D, Modified Rankin Scale scores were not different between patients with or without detectable CBA Abs, but in both cases mRS scores decreased at follow-up (FU) (2-tailed paired *t* tests). ^a^*P* = .003 for the change in mRS score at FU. ^b^*P* = .002 for the change in mRS score at FU.

All patient serum samples were studied together at the University of Oxford, Oxford, United Kingdom, for VGKC-complex antibodies by radioimmunoprecipitation and for LGI1, CASPR2, and contactin 2 antibodies by live cell–based assays (as used routinely in the University of Oxford clinical service). Eleven of the serum samples (29%) (9 from men and 2 from women)were VGKC-complex antibody positive. However, results of the live cell–based assays were positive in 17 of the 38 patients (45%), including CASPR2 antibodies in 11 (29%), LGI1 antibodies in 8 (21%), and contactin 2 antibodies in 5 (13%) (Figure 2A). Results from 8 of the 17 assays were positive for VGKC-complex antibodies by radioimmunoprecipitation, but high levels were only found in patients with both CASPR2 and LGI1 antibodies (Figure 2B). The antibodies occurred either singly or in combinations, and the live cell–based assay scores are shown in Figure 2C. Overall, antibody specificity did not influence mRS scores before or after treatments (Figure 2D and Table 2).

**Table 2.** Antibodies and Clinical Features in 38 Patients With Electromyography-Confirmed Neuromyotonia^a^

Antibody Finding	No. of Patients (No. M:F)	Clinical Feature, No. of Patients					Mean (SD) mRS Score Before/After Treatments	*P* Value
		Pain	Autonomic Features	Insomnia/Other Sleep Disturbance	Anxiety/Agitation/Depression	Additional Features or Abs		
Ab negative (n = 18) or VGKC-complex only (n = 3)	21 (14:7)	11	11	4/0	4/2/2	1 Thymoma, 1 prostate, 1 CRPS-like	2.92 (1.17)/1.25 (0.87) (n = 12)	.002
Specific Ab positive	17 (11:6)	9	7	4/4	3/3/2	4 Thymoma, 2 prostate, 2 AChR-Ab	2.9 (1.2)/1.8 (0.68) (n = 16)	.002
CASPR2 and LGI1	6 (5:1)	5	6	3/2	2/2/0	3 Thymoma (2 invasive, 1 AChR-Ab), 1 prostate, 1 limbic encephalitis	3.83 (1.17)/1.67 (0.52) (n = 6)	.002
CASPR2 (n = 4) or CASPR2 and contactin 2 (n = 1)	5 (1:4)	1	0	0/2	0/0/1	1 Thymoma, 1 prostate, 1 AChR-Ab MG	2.50 (1.29)/1.75 (0.96) (n = 4)	.39
Contactin 2 only	4 (3:1)	3	1	1/0	1/1/1	None	2.25 (0.50)/1.75 (0.96) (n = 4)	.39
LGI1 only	2 (2:0)	0	0	0/0	0/0/0	None	2.0/2.0^b^	NA

Abbreviations: Ab, antibody; AChR, acetylcholine receptor; CASPR2, contactin-associated protein 2; CRPS, complex regional pain syndrome; LGI1, leucine-rich glioma inactivated protein 1; MG, myasthenia gravis; mRS, modified Rankin Scale; NA, not applicable; VGKC, voltage-gated calcium channel.

^a^ Two-tailed *t* tests used that were not corrected for multiple comparisons. Numbers in the final column are limited to those with pretreatment and follow-up treatment mRS scores.

^b^ The SDs were not determined.

However, of the 6 patients with both CASPR2 and LGI1 antibodies, 3 (50%) had thymoma and 1 had developed prostate cancer; 1 had mild disease that responded to carbamazepine alone. Each of these patients had autonomic symptoms as well as typical NMT, 5 complained of pain, and 4 had neuropsychiatric features. Five had sleep disturbance and 3 fulfilled the criteria for Morvan syndrome (NMT, autonomic disturbance, and insomnia),^11^ although 1 had an additional central nervous system feature (seizures). As a group, they had higher mean pretreatment mRS scores (mean [SD], 3.83 [1.17]) compared with all other patients (2.71 [1.08]), but both groups had similar posttreatment scores (1.67 [0.52] vs 1.52 [0.88]; Table 2).

Serum samples were also tested simultaneously using a widely available commercial assay. Positivity for LGI1 and CASPR2 antibodies was confirmed except for 1 CASPR2 antibody that was not detected with the commercial assay (data not shown).

### Results of the Independent Patient-Led Pain Questionnaire

**Figure 3.**
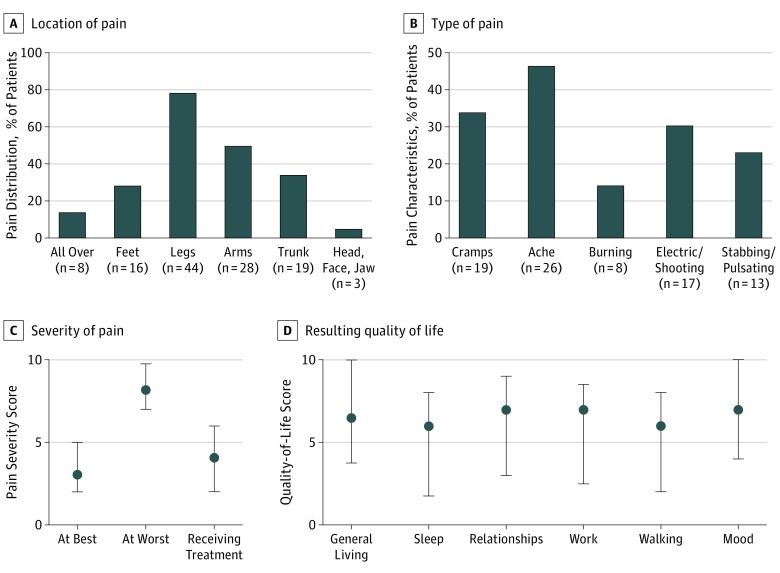
Characteristics of Pain in 56 Patients With Neuromyotonia (NMT) or Related Diagnoses A, Percentage of patients reporting pain in different anatomic regions. B, Percentage of patients reporting different types of pain. C, Pain scores at best, at worst, and after treatment. D, Influence of pain on quality of life. Patients responding to a patient-led online survey were asked to score their experiences of pain and its consequences on a scale of 0 to 10, with 0 indicating none or no effect and 10 indicating incapacitating or substantial effect. A summary of the questionnaire is in the Box, and the full questionnaire is provided in eTable 2 in the Supplement. Results in C and D are shown as median scores. Whiskers indicate interquartile range.

Because of the reports of pain in 20 patients (53%) that were often unrelated to muscle cramps, an independent survey was conducted via an Isaacs syndrome website, and questionnaires were sent to 165 individuals who reported pain. The patients were asked to describe the level of pain at best and at worst (0 representing no pain to 10 representing very bad or incapacitating pain), describe the nature of the pain, and detail where the pain was, what factors made it better or worse, and the extent to which it had responded to treatments. To assess the effects on quality of life, patients were asked the extent to which pain affected sleep, relationships, work, exercise, mood, and general activities (summarized in the Box and detailed in eTable 2 in the Supplement). Of the 56 of 165 individuals (34%) who returned detailed responses (32 males and 24 females; median [range] age, 50 [12-85] years) from different countries (mainly the United States, United Kingdom, and Australia), 8 had been given diagnoses of NMT, 28 of Isaacs syndrome, 4 of peripheral nerve hyperexcitability, 9 of cramp fasciculation syndrome, and 7, something else (1, Morvan; 1, cramps; 2, fibromyalgia; and 3, awaiting a diagnosis). The questions and scoring requested are summarized in the Box and detailed in eTable 2 in the Supplement. Pain was reported in the legs in 44 participants (79%) plus feet (16 [29%]) or arms (28 [50%]) but also in the trunk or neck (19 [34%]) (Figure 3A). Cramps and aching pain were the most common, but shooting or “electric” pain were noted by 17 (30%) and stabbing or pulsating pain by 13 (23%) (Figure 3B); burning pain was reported by 8 participants (14%). Most patients experienced intermittent pain, with scores differing substantially between “at best” and “at worst,” and treatments appeared helpful (Figure 3C). In many patients, pain had, or previously had, a major influence on aspects of daily living (Figure 3D).

Box. **Summary of Pain Questionnaire Sent to Individuals Responding to a Patient-Led Online Survey^a^**

Country, sex, age, medical diagnosis (eg, NMT, Isaacs syndrome, peripheral nerve hyperexcitability, CFS, or other).
How bad is the pain at best (1 = little pain, 10 = very painful-incapacitating)?
How bad is the pain at worst (1 = little pain, 10 = very painful-incapacitating)?
How would you describe the types of pain(s) you feel, and where on the body they occur? Please also add how bad is/are the pain(s) in each area.
What if anything make the pain(s) worse (eg, temperature changes, any form of exercise or exertion, stress, or any foods)?
When do your pain symptoms occur (eg, in the morning/evening, any time of day, intermittent, or constant)?
How effective have your prescriptions been at helping with pain(s) (1 = totally ineffective, 10 = fully effective)?
For the following questions, please select between 1 and 10 as appropriate (1 = has had little effect, 10 = affects sleep very much).
How much would you say pain affects your enjoyment of life?
How much would you say the pain(s) affects sleep?
How much would you say pain affects your relations at /home/with loved ones/friends?
How much would you say pain affects your ability to work (includes work around the home)?
How much would you say pain affects your walking ability?
How much would you say pain affects your mood?
How much would you say pain affects overall day-to-day living and general activities?
Finally, if you wish, comment further about the pain(s) you suffer and how they affect you.

Abbreviations: CFS, cramp fasciculation syndrome; NMT, neuromyotonia.

^a^This is a simplified version of the form sent to patients who responded to the internet survey as having pain. The full form, which includes example answers for each question, is available in eTable 1 in the Supplement[Boxed-text noi180062b1]. The results were entered into a spreadsheet file after deidentification by one of us (R.B.) and analyzed by another of us (A.V.).

## Discussion

There have been few detailed descriptions of NMT,^14,15,16,20^ and none have studied in detail the associated clinical features, serologic characterization, and treatment responses. The results of this cohort study of 38 patients with EMG-defined NMT found 8 (21%) with concurrent tumors (5 thymoma, 3 prostate); when asked, a high proportion reported additional features of autonomic disturbance, pain, and central nervous system dysfunction. Antibodies to the now well-recognized VGKC-complex antigens were present in 17 of the 38 patients (45%), but these included 6 (16%) patients with both LGI1 and CASPR2 antibodies, and these patients had more severe disease, thymoma (in 50%), and features of Morvan syndrome. Contactin 2 antibodies were relatively common (5 patients [13%]) compared with their incidence in other VGKC-complex antibody disorders.^8^ After receiving treatment, all but 4 patients with follow-up data improved by at least 1 mRS score. Therapies to treat symptoms were moderately effective in the few patients with relatively mild disease; however, in others, improvement was related to immunotherapy with or without symptom treatments.

The high frequency of pain among the patients with EMG-defined NMT (20 patients [53%]) exceeded that reported in previous studies, and the nature of the pain in NMT has only occasionally been described^13,22,23^ and may previously have been ascribed mainly to muscle cramps. However, the study patients with NMT reported a variety of painful symptoms as reflected in the detailed responses reported via the independent, patient-led online questionnaire that included fuller descriptions of the pain, its distribution and effects on aspects of daily living, such as relationships and work. A systematic, prospective study of pain in NMT needs to be undertaken.

Another feature of this study was the number of patients who reported autonomic features. These have always been recognized in NMT,^3,13^ but direct questioning resulted in almost 50% of both male and female patients reporting diverse symptoms. In addition, sleep disturbance, including insomnia, were frequent. Morvan syndrome, which is defined by NMT, autonomic disturbance, and insomnia with encephalopathy, is thought to be a rare disease, with fewer than 100 cases reported in the literature.^10,11,24^ One typical patient with Morvan syndrome and high-titer CASPR2 antibodies was not included here because the diagnosis had already been given and serum was no longer available. Nevertheless, 3 of the 6 patients (50%) with both CASPR2 and LGI1 antibodies had many features of Morvan syndrome, including thymoma; however, none had developed encephalopathy, and the diagnosis remained NMT. Thus, NMT can be seen as a forme fruste of Morvan syndrome.

In this study, 3 of the patients had VGKC-complex antibodies without evidence of positivity for CASPR2, LGI1, or contactin 2. Although VGKC-complex antibodies can be at high titer (>400pM)^8^ in patients with NMT, the titers originally reported^3,13^ were mostly lower (<400pM), and in the present study, 47% of patients were negative for all antibody tests (<100pM, the cutoff used at the University of Oxford; other centers with lower cutoffs may calculate titers differently). In fact, the clinical relevance of many lower titers, often without evidence of antibodies to LGI1, CASPR2, or contactin 2, is unclear.^25,26^ By contrast, LGI1 antibodies modulate VGKC function on brain slices and α-amino-3-hydroxy-5-methyl-4-isoxazolepropionic acid currents in hippocampal neurons^27,28^ and internalize the LGI1–disintegrin and metalloproteinase 22 receptor complex in transfected human embryonic kidney cells.^29^ CASPR2 antibodies did not internalize CASPR2 on hippocampal neurons in one study,^30^ but in vivo transfer of CASPR2-antibodies to mice produced afferent nerve hyperexcitability and mechanical allodynia with loss of surface CASPR2 and VGKC Kv1 subunits in the dorsal root ganglia; these and additional dorsal horn changes were found in CASPR2^−/−^ mice.^18^ These findings are typical of neuropathic pain models and consistent with a contribution of CASPR2 antibodies to producing pain via an effect at the levels of the dorsal root in NMT. Autoimmunity in patients with pain is an emerging field of research.^31^

The data here and from other studies^17,22,23^ suggest that CASPR2, LGI1, and contactin 2 antibodies are more relevant than VGKC-complex antibodies in patients with suspected NMT. Nevertheless, fewer than half of the patients with suspected acquired autoimmune NMT were positive for the antibodies tested here, and other targets may need to be identified. At present, neurophysiologic assessment remains the criterion standard for diagnosis combined with clinical phenotyping.

Treatment protocols for NMT have not been formalized. Most patients will be given antiepileptic therapies or muscle relaxants as a first-line treatment because these can be sufficient; in the present series, these therapies were most often prescribed for patients with less severe disability. The remaining patients were given these treatments plus corticosteroids, immunotherapies, or both. Such therapies were effective and most patients reported benefit, but some patients noted little benefit. In patients with symptoms of NMT refractory to immunotherapies, the possibility of nonimmunologic disorders should be considered.

### Limitations

Neuromyotonia is a rare disorder and consequently study numbers tend not to be large. Furthermore, as a study driven by patients and their clinical needs rather than through an established protocol, data were typically collected at follow-up reviews. As such, it was not always possible to obtain mRS scores from all patients before and after treatment. Serologic studies of LGI1, CASPR2, and contactin-2 antibodies were performed on archived samples that had been sent to the Oxford reference laboratory as part of routine diagnostic screening from Sydney and Kagoshima and were then retested for this study. Separately, although there are limitations with relying on a patient-initiated questionnaire—including a lack of response in those patients who did not report pain—it is increasingly recognized that patient-reported outcome measures may better reflect real-world experiences, which in this case have provided novel insight into the variable frequency and characteristics of pain in patients diagnosed with NMT and on the effects on their daily activities.

### Conclusions

Although cramps, muscle fasciculations, and pain are common symptoms, neuromyotonia remains a relatively rare diagnosis, and it is likely that less severe cases are often unrecognized. Nevertheless, comorbidities such as thymoma or myasthenia and sometimes associated symptoms of autonomic and central nervous system disturbance, and pain, a potentially disabling feature that is not always appreciated in clinical practice, mean that the disease deserves to be investigated for underlying triggers while also treated appropriately. Serologic studies are not positive in all patients, and neurophysiology remains a key diagnostic tool that also serves as a means of phenotyping patients. Moreover, although heterogeneity of clinical features and related antibodies may limit interpretation of significant correlations, the coexistence of LGI1 and CASPR2 antibodies may suggest the presence of thymoma, often accompanied by autonomic and central nervous system involvement.

## Notes

Supplement. **eTable 1.** Clinical Datasheet **eTable 2.** Patient-led On-line Questionnaire as Sent to 165 Individual Patients Who Reported Pain **eTable 3.** Other Autoimmunity or Co-morbidities as Reported by Clinicians **eTable 4.** Responses to Different Treatments
