# Supplementary material for: Association of Leucine-Rich Glioma Inactivated Protein 1, Contactin-Associated Protein 2, and Contactin 2 Antibodies With Clinical Features and Patient-Reported Pain in Acquired Neuromyotonia
Source: JAMA Neurol. 2018 Sep 17;75(12):1519–27. doi: 10.1001/jamaneurol.2018.2681 (PMC6583195; doi:10.1001/jamaneurol.2018.2681)
Supplement: Supplement. — eTable 1. Clinical Datasheet eTable 2. Patient-led On-line Questionnaire as Sent to 165 Individual Patients Who Reported Pain eTable 3. Other Autoimmunity or Co-morbidities as Reported by Clinicians eTable 4. Responses to Different Treatments [file jamaneurol-75-1519-s001.pdf]

Supplementary Online Content

Vincent A, Pettingill P, Pettingill R, et al. Association of leucine-rich glioma inactivated protein 1, contactin-associated protein 2, and contactin 2 antibodies with clinical features and patient-reported pain in acquired neuromyotonia. *JAMA Neurol*. Published online September 17, 2018. doi:10.1001/jamaneurol.2018.2681

eTable 1. Clinical Datasheet

eTable 2. Patient-Led On-line Questionnaire as Sent to 165 Individual Patients Who Reported Pain

eTable 3. Other Autoimmunity or Co-morbidities as Reported by Clinicians

eTable 4. Responses to Different Treatments

This supplementary material has been provided by the authors to give readers additional information about their work.

eTable 1. Clinical datasheet

Patient's code…………………………; Age ; Sex: M / F

Tumour diagnosed? Y / N. If yes, which tumour?..

Days from neurological symptoms until tumour diagnosis…………………………

Duration at time of serum sampling? ……………………………………….

Was sampling before any immunological treatments?......................................

	Please answer questions and provide furth details/comments
**First symptom(s)**	
**Associated infection or other precipitant (prece or concurrent)**	
**Sweating?**	
**Cramps?**	
**Twitching?**	
**Pseudomyotonia?**	
**Stiffness or weakness?**	
**EMG/NCS results**<br>**-Multiplets or doublets?**<br>**-High frequency bursts (eg 400/sec)?**<br>**-Conduction slowing or other evidence of neuropathy?**	
**Pain, paraesthesias or other sensory symptoms??**<br><br>**Please describe pain if possible – ie burning, sho throbbing etc**	
**Autonomic disturbance - please detail any**<br><br>**Tachycardia?**<br><br>**Constipation?**<br><br>**Excessive secretions?**<br><br>**Urinary symptoms?**	

**Sleep disturbance?**<br><br>**Insomnia?**<br><br>**Hypersomnia?**<br><br>**REM-SBD?**	
**Mild psychiatric features? Eg. increased anxiety agitation?**	
**More prominent symptoms? eg. confusion/ disorientation/ amnesia / psychosis /depression?**	
**Seizures (please note semiology and frequency)?**<br><br>**Any other neurological disease?**	
**Past/current medical history**<br><br>**Hypertension, diabetes, other autoimmune disor**<br><br>**Relevant family history?**	
**MRI, EEG and CSF results if performed**	
**Tumour?**<br><br>**Whole body imaging results**	
**Other clinical symptoms, signs or test results of relevance?**	
**Severity, treatment response and outcome**	
**How disabling were the symptoms?**<br><br>**Please give the modified Rankin Scale score (see below) at nadir***	
**What treatments were tried and were they effect**<br><br>**Phenytoin**<br><br>**Carbamezepine**<br><br>**Steroids**<br><br>**Plasma exchange**<br><br>**Intravenous immunoglobulins**<br><br>**Others**	

**Residual clinical features and post-treatment modified Rankin Scale score***	

Modified Rankin Scale scores

0. Asymptomatic patient;

1. Symptoms do not interfere with lifestyle;

2. Symptoms lead to some restriction of lifestyle but do not prevent totally independent existence;

3. Symptoms significantly interfere with lifestyle or prevent totally independent existence;

4. Symptoms prevent independent existence, but patient does not need constant attention day & night;

5. Severe disability, with patient totally dependent and requiring constant attention day and night;

6. Death due to the encephalopathy (modified Rankin scale from Graus et al 2001, Brain 124;1138-48)

eTable 2. Patient-led on-line questionnaire as sent to 165 individual patients who reported pain

Country
Sex
Age
What is your medical diagnosis?<br>Have you been diagnosed with Isaacs / Syndrome /CFS / PNH or any other related disease?
For the following questions please select between 1 and 10 as appropriate.
How bad is the pain at best (1=little pain, 10=very painful-incapacitating)? Eg. At best my pain is 6.
How bad is the pain at worst (1=little pain, 10=Very painful-incapacitating)? Eg. At worst my pain is 7.
What if anything make the pain/s worse (eg temperature changes, any form of exercise or exertion, stress, any foods)? Eg. leg pain is worse after exercise.
When do your pain symptoms occur (eg. in the morning/evening, any time of day, intermittent, all the time, constant)? Eg. Aches in leg muscles in the morning, arm pains in the evening.
How would you describe the types of pain/s you feel and where on the body they occur (eg aches in leg muscles, pulsating pains in legs, shooting pains allover, stabbing pain in neck etc)? If you have more than one pain type and or body area/issue please do list and describe them all.<br>Please also add how bad is/are the pain/s. Eg. Pulsating leg pain 7, arm muscle ache 4, body ache 9, shooting pains allover 7.
How effective have your prescriptions been at helping with pain/s (1=totally non-effective, 10=fully effective)? Eg. For my leg pain 5. For my arm pain 3.…etc.
How much would you say pain affects your enjoyment of life (1=has had little effect, 10=very much negatively affects the enjoyment of my life)? Eg. my enjoyment has been affected quite badly, I can’t do this or that anymore 7.
How much would you say the Pain/s affects sleep (1=has had little effect, 10=affects sleep very much)? Eg. I dont sleep as deeply and I wake up early, really affects my day 8.
How much would you say pain affects your relations work/home/loved ones/friends (1=has had little negative effect, 10=very much negatively affects)? Eg. I dont go out as much to see friends/relatives when the pain is at its worst, but sometimes I can 6.
How much would you say pain affects your ability to work (includes work around the home; 1=has had little effect, 10=very much negatively affects my work life)? Eg. I am only now able to work part time 7.
How much would you say pain affects your walking ability (1=has had little effect, 10=very much affects my ability to walk around)? Eg. I can only get around with a stick or a walker 8.
How much would you say pain affects your mood (1=has had little effect, 10=very much affects my mood)? Eg. in the morning when the pain is bad its 8, better during the daytime 3.
How much would you say pain affects overall day to day living - general activities (1=has had little effect, 10=very much negatively affects doing things I have to or

want to do)? Eg. I can't go shopping on my own and need help picking up the kids 6.
Finally, this is the place where you can comment further about the pains you suffer and how they affect you.<br><br>Thank you very much for taking part in this first survey!

eTable 3. Other autoimmunity or co-morbidities as reported by clinicians

Males	Females
AGA susp, asthma, sister and son have cramps	Bronchiectasis, ulcerative colitis, multiple pulmonary embolism after haemoptysis
Degenerative disc, father twitching, mother TIID	Cardiovascular disease
1 diabetes, 1 FH diabetes	1 hyperparathyroidism with low vitamin D, eczema, iron deficiency, sister with Crohn's disease
3 hypertension	1 IgA nephropathy, hypothyroidism
Leukaemia, acute VGHD	1 systemic lupus erythematosus
Lung cancer, diabetes, malignant hyperthermia	
FH motor neuron disease	
1 ocular MG AChR Ab	
Recurrent urinary tract infections, hypogammaglobulinaemia, chronic fatigue syndrome, FH diabetes	
Past history renal failure, previous red cell aplasia	
Spondylosis, uncle neurological mother and brother nephritis	
Thalassaemia	
1 AChR, 2 ANA	3 AChRs, 3 ANAs

eTable 4. Responses to different treatments

	Total	Males	Females
**Treatments (no of patients)**			
AED good response	15	7	8
AED poor response	2	1	1
Good response to steroids	6	4	2
Poor or no response	6	4	2
Good response to IvIg, FK506, CysA or rituximab	14	10	4
Poor or no response	3	3	0
Good response to plasma exchange	6	3	3
Poor or no response	2	0	2
